# CNDP1, NOS3, and MnSOD Polymorphisms as Risk Factors for Diabetic Nephropathy among Type 2 Diabetic Patients in Malaysia

**DOI:** 10.1155/2019/8736215

**Published:** 2019-01-03

**Authors:** Mohd Jokha Yahya, Patimah Binti Ismail, Norshariza Binti Nordin, Abdah Binti Md Akim, Wan Shaariah Binti Md Yusuf, Noor Lita Binti Adam, Nurul Fasihah Zulkifli

**Affiliations:** ^1^Department of Biomedical, Faculty of Medicine and Health Sciences, Universiti Putra Malaysia, Seri Kembangan, Malaysia; ^2^Department of Human Development and Growth, Faculty of Medicine and Health Sciences, Universiti Putra Malaysia, Seri Kembangan, Malaysia; ^3^Department of Medicine, Department of Medicine (Endocrinology & Nephrology), Hospital Tuanku Ja'afar, Seremban, Malaysia

## Abstract

Type 2 diabetes mellitus (T2DM) is associated with a high incidence of nephropathy. The aim of this study was to investigate the association of a genetic polymorphism of carnosinase (*CNDP1-D18S880* and -*rs2346061*), endothelial nitric oxide synthase (*NOS3-rs1799983*), and manganese superoxide dismutase (*MnSOD-rs4880*) genes with the development of diabetic nephropathy among Malaysian type 2 diabetic patients. A case-control association study was performed using 652 T2DM patients comprising 227 Malays (without nephropathy = 96 and nephropathy = 131), 203 Chinese (without nephropathy = 95 and nephropathy = 108), and 222 Indians (without nephropathy = 136 and nephropathy = 86). DNA sequencing was performed for the *D18S880* of *CNDP1*, while the rest were tested using DNA Sequenom MassARRAY to identify the polymorphisms. DNA was extracted from the secondary blood samples taken from the T2DM patients. The alleles and genotypes were tested using four genetic models, and the best mode of inheritance was chosen based on the least *p* value. The *rs2346061* of *CNDP1* was significantly associated with diabetic nephropathy among the Indians only with OR = 1.94 and 95% CI = (1.76–3.20) and fitted best the multiplicative model, while *D18S880* was associated among all the three major races with the Malays having the strongest association with OR = 2.46 and 95% CI = (1.48–4.10), Chinese with OR = 2.26 and 95% CI = (1.34–3.83), and Indians with OR = 1.77 and 95% CI = (1.18–2.65) in the genotypic multiplicative model. The best mode of inheritance for both *MnSOD* and *NOS3* was the additive model. For *MnSOD*-*rs4880*, the Chinese had OR = 2.8 and 95% CI = (0.53–14.94), Indians had OR = 2.4 and 95% CI = (0.69–2.84), and Malays had OR = 2.16 and 95% CI = (0.54–8.65), while for *NOS3*-*rs1799983*, the Indians had the highest risk with OR = 3.16 and 95% CI = (0.52–17.56), followed by the Chinese with OR = 3.55 and 95% CI = (0.36–35.03) and the Malays with OR = 2.89 and 95% CI = (0.29–28.32). The four oxidative stress-related polymorphisms have significant effects on the development of nephropathy in type 2 diabetes patients. The genes may, therefore, be considered as risk factors for Malaysian subjects who are predisposed to T2DM nephropathy.

## 1. Introduction

The global obesity rates have reached an epidemic proportion [1] the same way diabetes mellitus (DM) has become a global health problem. The continuously changing lifestyles which have led to reduced physical activity and increased obesity are mainly responsible for the significant increase in numbers of DM patients in nearly all countries [2]. In Malaysia, the dramatic prevalence leap for diabetes from 11.6% in 2006 [3] to 22.9% in 2011 [4] has alarmingly exceeded the postulated level (13.4%) for the population for 2025 by the World Health Organization diagnostic criteria [2].

One of the microvascular complications of DM is diabetic nephropathy (DN) or diabetic kidney disease, a syndrome characterized by the presence of pathological quantities of urine albumin excretion, diabetic glomerular lesions, and loss of glomerular filtration rate (GFR) in diabetics [5, 6]. Unfortunately, detecting the early symptoms of nephropathy is almost impossible in diabetic patients particularly when the onset of diabetes is unknown. Thus, this may result in poor early patient management and eventually cause rapid kidney deterioration. Usually, most patients are unaware of being diabetic and nephropathy has already manifested when they are diagnosed. The exact etiology or biochemical pathology of type 2 diabetes mellitus (T2DM) nephropathy is impossible to be determined immediately with the simple existing tests.

Furthermore, 91% of diabetic patients are diagnosed or have been diagnosed as diabetic nephropathy, and 50% of dialysis patients are a result of T2DM complications [7, 8]. The rise of the T2DM is due to an increase in overweight and obesity factors as a result of an unhealthy lifestyle. Various strategies have thus been established by the Malaysian government to overcome the increasing number of diabetes cases by focusing particularly on healthy lifestyle promotions and screening for diabetes and complications in the population [9].

Dyslipidemia, hypertension, and glycemic control are generally modifiable risk factors for most chronic diseases, while the main unmodifiable risk factors are age, race, and genetic profile [10–12]. Therefore, DN is more likely to develop in patients with a family history of DN. A meta-analysis has also identified 24 genetic variants in 16 genes as being associated with DN [5]. T2DM has also become the most prevalent metabolic disease globally being a multifactorial disorder as a result of the interaction of the environmental factors with the individual's genetic background [13]. Some common examples of genes which link oxidative stress to diabetic nephropathy are carnosinase, endothelial nitric oxide synthase, and manganese superoxide dismutase genes.

Carnosinase dipeptidase 1 (*CNDP1*) and carnosinase dipeptidase 2 (*CNDP2*) genes lie adjacently in chromosome 18 at 18q22.3. *CNDP1* encodes for carnosinase dipeptidase 1 that specifically degrades carnosine to *β*-alanine and L-histidine in the serum, while *CNDP2* encodes for a nonspecific carnosinase in the tissue [14]. Carnosine (*β*-alanyl-L-histidine) acts as a protector to renal against nephropathy among diabetic patients by scavenging oxygen species. One of the major causes of vascular pathology in diabetes, however, is mitochondrial overproduction of ROS [15]. Thus, carnosine helps to lower ROS levels which inhibit the formation of AGEs and reduce production of TGF*β*, thereby preventing the risk of nephropathy among the T2DM patients [16]. Another polymorphism that had shown association with nephropathy is at the promoter region of *CNDP1* identified as *rs2346061* [17].

NO is an important product of endothelium cells synthesized by the enzymatic reaction and catalyzed by the endothelial nitric oxide synthase (eNOS) from L-arginine. eNOS is expressed by the *eNOS* gene which is located in chromosome 7q35-q36 and contains 26 exons with a total length of 21 kB [18]. The three polymorphisms found to be associated with diabetic nephropathy susceptibility are gene *G894T* missense mutation (*rs1799983*), a 27 bp repeat in intron 4, and the *T786C* single-nucleotide polymorphism (SNP) in the promoter (*rs2070744*). Despite some contradictions, *G894T* (*rs1799983*) is the most common polymorphism to have an association with T2DM nephropathy, especially among the East Asians [19]. Polymorphism of *G894T* causes a missense substitution in exon 7 which modifies the activity of eNOS.

Manganese superoxide dismutase (MnSOD), also known as superoxide dismutase 2 (SOD2), is a mitochondrial protein. MnSOD is expressed by its genes which are located in chromosome 6q25. The SNP C to T, C47T, is registered as *rs4880* in exon 2, resulting in MnSOD with an amino acid substitution of valine with alanine at position 16 (Val16Ala). MnSOD protects cells from oxidative stress that can cause damage by scavenging free radicals. The genetic variation of *rs4880* SNP has been associated with diabetes and some of its complications such as cardiovascular disease, nephropathy, neuropathy, and retinopathy [20, 21].

The aim of this study was, therefore, to investigate the association of a genetic polymorphism of *CNDP1-D18S880* and -*rs2346061*, *NOS3*-*rs1799983*, and *MnSOD*-*rs4880* genes with the development of diabetic nephropathy among Malaysian type 2 diabetic patients.

## 2. Materials and Methods

A case-control association study between cases with nephropathy and controls without nephropathy of T2DM patients was used. The study conforms to the items of the Declaration of Helsinki and was approved by the National Medical Research Register (NMRR) of Ministry of Health Malaysia (KKM) with reference number (2) DLM.KKM/NIHSEC/08/0804/P12-519. A written and signed informed consent was obtained from all the nephrotic T2DM subjects (cases), while consent forms were also obtained from all of the nonnephrotic T2DM subjects (controls) who enrolled in the current study. This study was carried out on T2DM Malaysian subjects from three major ethnic groups, namely, Malays, Chinese, and Indians. Both case and control subjects were recruited from the outpatients of the Medical Clinic of Hospital Tuanku Ja'afar Seremban (HTJS), Negeri Sembilan, Malaysia. Patients were from nearby states such as Melaka, Negeri Sembilan, Southern Selangor, and also Kuala Lumpur.

### 2.1. Sampling Method

More than 1000 T2DM patients' records were screened from the medical outpatient department in HTJS. A total of 820 were found to be suitable and available for the research, but only 652 were actually tested belonging to the three different ethnic groups in Malaysia: the Chinese, 203 samples; Malays, 227 samples; and Indians, 222 samples. Either the remaining 178 individuals were not interested to be part of the research or the quality of the blood samples collected or the DNA extracted was compromised. The selection of the samples was based on the inclusion and exclusion criteria for nephropathy (case) and nonnephropathy (control) as shown in Table 1 and also on expert opinions of the specialists in the endocrinology and nephrology clinics. Only interested patients with signed consents were allowed to participate in this research. Patients were allowed to withdraw from the research if they wanted to.

### 2.2. DNA Extraction and Genotyping

The blood samples used were secondary samples taken from the laboratory tested for HbA1C for T2DM patients who came for their routine visits. The samples were with ethylenediamine tetraacetic acid (EDTA) anticoagulant Vacutainer tubes and stored at −20°C for DNA extraction and analysis. Patients' most recent biochemical results were obtained from the laboratory information system (LIS) in the Department of Pathology. The laboratory is accredited and certified under MS ISO 15189. The patients' demography was obtained from the patients' records. Genomic DNA was extracted using a commercial DNA extraction kit (QIAGEN, USA). DNA genotyping included the DNA extraction, quantification, impurity testing, and amplification. The SNP genotyping was carried out using the Sequenom MassARRAY iPLEX platform, while sequence analysis was done using Sanger sequencing to detect the five CTG repeats on *CNDP1*-*D18S880* only. The primer was derived by Ahluwalia et al. [17]. The sequence of primers and size of the PCR products used for the genotyping are listed in Table 2.

### 2.3. Statistical Analysis

All statistical genetic analyses were performed using the Statistical Package for the Social Sciences version 17.0 (SPSS v17.0). The frequencies of alleles and genotypes were studied by means of descriptive and inferential statistics to compare between cases and controls. One-way analysis of variance (ANOVA) was used to test the differences between the clinical data. Pearson's chi-square goodness-of-fit test was used to test whether the controls were in a Hardy–Weinberg equilibrium (HWE) and also to test the distribution of the variants. The genotype frequencies for each SNP were tested using conventional Pearson's chi-square test for independence with df = 2 to determine the association with T2DM nephropathy. A *p* < 0.05 (two-tailed) was considered as the criterion of statistical significance. The Fisher exact test 2 × 3 was performed when more than 20% of the cells had expected values less than 5, while test 2 × 2 was performed when more than 20% of the cells had expected values less than 10. The significant SNPs were further tested using 3 types of the model using chi-square with df = 1. Further testing was done using the Cochran–Armitage trend test. The strength of association or the risk of developing diabetic nephropathy was determined by the odds ratio (OR) with corresponding 95% confidence interval (CI). The OR is calculated using VassarStats, an online statistical computation website. The mode of inheritance was determined by choosing the model with the least *p* value.

## 3. Results

### 3.1. Characteristics of T2DM Sampling Subjects

The subjects comprised 227 Malays, 203 Chinese, and 222 Indians from a total of 652 T2DM patients. The number of samples was almost evenly distributed among the three races, although the figures do not reflect the true sociodemographic distribution of the ethnics in the population. The number of samples was to fulfill the requirement for the statistical analysis. The age range of the Malay T2DM subjects was between 32 and 83 years, with a mean of 59.0 ± 8.23; the Chinese was between 36 and 89 years, with a mean of 63.28 ± 11.56; and the Indians was between 35 and 86 years, with a mean of 61.33 ± 10.1. A majority of the subjects were aged between 58 and 64 years (77.5%). Furthermore, there were 121 (53.3%) Malay males and 106 (46.7%) females; 107 (52.7%) Chinese males and 96 (47.3%) females; and 113 (50.9%) Indian males and 109 (49.1%) females. T2DM nephropathy patients were selected basically by the AER more than 300 mg/24 h. Samples with less than 300 mg/24 h AER were not considered. Patients were not staged according to the development of nephropathy as the aim of this study was to observe the effects of polymorphism as risk factors. The patients' biochemical level retrieved from the laboratory information system was to avoid bias in the sampling. The biochemistry of the patients is described in Table 3.

### 3.2. Demographic Characteristics of the Study Subjects

The clinical demographics of the subjects in this study are shown in Table 3. Although all the races showed similar characteristics, there were, however, significant differences (*p* < 0.05) between cases and controls in AER, total cholesterol, and HDL. However, no significant difference (*p* > 0.05) was observed in the duration of diabetes, glycated hemoglobin concentration, fasting blood glucose, LDL, and triglycerides level in the T2DM cases and controls. There was a significant difference (*p* < 0.01) only in the glycated hemoglobin (HbA1C) concentration and glycemic control in comparison of the biochemical parameters among the races using one-way ANOVA (data not shown). The Chinese, however, had the worst glycemic control in both cases and controls.

### 3.3. Genotyping of Polymorphisms

There were only two variables: 5CTG and 6CTG repeats, found in the entire population, and the distribution of the genotype was in patterns of 5-5, 5-6, and 6-6. The other polymorphisms were tested by the MassARRAY, and the homozygous and heterozygous genotypes were interpreted by the observation of peaks produced in the chromatogram. The results are shown in Table 4 (Supplementary Materials (available here)).

### 3.4. Chi-Square Test of Genotype and Allele Association

In this study, all controls were tested for HWE, and the results showed that all the controls were in the HWE with *p* > 0.05 as shown in Table 5. For the carnosinase gene (*CNDP1*), *D18S880* genotype associated with nephropathy in the Malays (*p*=0.026), Chinese (*p*=0.0171), and Indians (*p*=0.0095) (Table 6), as supported by the alleles in Malays (*p*=0.0004), Chinese (*p*=0.0019), and Indians (*p*=0.0055). On the contrary, for *rs2346061* of the same gene, only the Indians showed association (*p*=0.0488). The *rs4880* showed genotypic association among the Malays (*p*=0.0450), Chinese (*p*=0.0380), and Indians (*p*=49.2 × 10^−5^) with nephropathy. As for *rs1799983*, it showed significant association of genotypes among the Malays (*p*=0.0158), Chinese (*p*=0.0146), and Indians (*p*=0.6174). The *rs1799987* genotype was observed to have a significant difference in the Chinese only (*p*=7.4 × 10^−5^), and *rs4073* has a significant difference in the Indians only; for the genotype (*p*=0.0200), the Indians (*p*=0.0047) and Chinese (*p*=0.0033) showed a genotypic significant difference for *rs17576* but the Malays did not. The allele had a significant difference among the Chinese (*p*=0.0013) and Indians (*p*=0.0033).

### 3.5. Dominant and Recessive Models

The association of the genotypes was then stratified against the dominant and recessive models as shown in Table 6. The genotypic variants showed a significant association of nephropathy with the *D18S880* recessive model among Malays (*p*=0.0006), Chinese (*p*=0.0075), and Indians (*p*=0.0132). On the contrary, the dominant model did not show any association among the Malays (*p*=0.1302) and Chinese (*p*=0.0540) but not among the Indians (*p*=0.0132), as in DN as compared in cases and controls. The *rs17576* showed an association with nephropathy among the Chinese (*p*=0.002) and Indians (*p*=0.0169) using the recessive model. In the dominant model, the Chinese (*p*=0.0221) showed an association, but the Indians did not (*p*=0.0512). Meanwhile, *rs1799987* showed significant association in both dominant (*p*=9.2 × 10^−5^) and recessive (*p*=0.0021) models among the Chinese only. As for *rs4073*, it did not show any association in both recessive (*p*=0.678) and dominant (*p*=0.876) models among the Indians.

### 3.6. Cochran–Armitage's Trend Test

For best understanding of the mode of inheritance and to calculate the additive model association, the association was tested for the trend using Cochran–Armitage's trend test (C-ATT). From Table 7, it can be seen that the best model fit for *D18S880* was multiplicative for all the races: Malays (*p*=0.0004), Chinese (*p*=0.0019), and Indians (*p*=0.005). For other variants, the best model was the additive. The best model was chosen based on the least *p* value compared to the other models.

The *D18S880* showed that the variants (6CTG) behaved dominantly (*p*=0.0006) than recessively (*p*=0.1309), but by using C-ATT, the variant behaved more towards being multiplicative with *p*=0.0004. The same model behavior could be observed in Chinese and Indians. The same was observed for *rs2346061* and the mode of inheritance that best suited was multiplicative with *p*=0.0396, while the other models were not showing association with nephropathy. As for the rest of the variants, the model that best suited the association was an additive model. The polymorphisms obeyed the additive genetic model and increased the risk of *r* for a variant *v* and 2*r* for *vv*. The characteristics could be observed in the odds ratio calculated as in Table 7.

### 3.7. Strength of Association

It was observed that *rs2346061* was associated with the Indians only. The best mode of inheritance is multiplicative with OR = 1.94 and 95% CI = (1.76–3.20) tested with the Cochran–Armitage trend. For the *CNDP1-D18S880* among the three races, the Malays had the strongest association with OR = 2.46 and 95% CI = (1.48–4.10), followed by the Chinese with OR = 2.26 and 95% CI = (1.34–3.83) and the Indians with OR = 1.70 and 95% CI = (1.18–2.65), as in the genotypic multiplicative model. This could be observed from Table 7 where the Malays showed the strongest association of variants towards nephropathy in T2DM patients. The same could be observed in the recessive model, in Malays with OR = 2.76 and 95% CI = (1.53–4.99), Indians with OR = 1.25 and 95% CI = (0.65–2.40), and Chinese with OR = 1.07 and 95% CI = (0.63–1.83).

The recessive model does not represent *D18S880* association well due to the weaker strength of the association shown. On the contrary, good representation of OR could be observed in the dominant model among Malays with OR = 2.84 and 95% CI = (1.16–3.75) and Indians with OR = 3.74 and 95% CI = (1.24–11.32) but not for the Chinese where OR = 1.90 and 95% CI = (0.49–7.39). The additive model was not applicable to this variant as the chi value was negative.

For the *MnSOD-rs4880* variant, the best model that fits the association was additive to the Malays having OR = 1.037 and 95% CI = (0.24–4.43) per copy of T and OR = 2.16 and 95% CI = (0.54–8.65) as the copy doubled. The Chinese showed a strong association in the additive model with OR = 1.28 and 95% CI = (0.23–7.25) per copy of T and OR = 2.8 and 95% CI = (0.53–14.94) as the copy doubled. Meanwhile, the Indians additive model showed association strength with OR = 1.16 and 95% CI = (0.32–4.23) per copy of T and OR = 2.4 and 95% CI = (0.69–8.24) as the copy doubled. From Table 7, it can be seen that the Chinese have the strongest association of *rs4880* followed by the Indians and Malays. The recessive model showed that the Indians have high association with OR = 2.77 and 95% CI = (1.49–5.15), followed by the Chinese with OR = 2.26 and 95% CI = (0.43–11.92) and the Malays with OR = 1.75 and 95% CI = (0.44–6.95). For the multiplicative model, Malay subjects with OR = 1.86 and 95% CI = (1.12–3.08) have the weakest association compared to the Chinese with OR = 2.02 and 95% CI = (1.17–3.47) and Indians with OR = 2.22 and 95% CI = (1.50–3.28).

For the *NOS3-rs1799983*, however, the additive model has the least *p* value for the association of nephropathy and T2DM, compared to the other models in this variant. Thus, it is the best model to represent the variant behavior. The Chinese showed the strongest association with OR = 1.60 and 95% CI = (0.16–16.23) per copy of T and OR = 3.55 and 95% CI = (0.36–35.03) for double copy of T, compared to the Indians with OR = 1.46 and 95% CI = (0.23–9.27) per copy of T and OR = 3.16 and 95% CI = (0.52–17.56) for double copy of T and the Malays with OR = 1.33 and 95% CI = (0.13–13.48) per copy of T and OR = 2.89 and 95% CI = (0.29–28.32) for double copy of T. The Chinese also showed the strongest association with nephropathy development for this polymorphism observed in the recessive model with OR = 2.71 and 95% CI = (0.28–26.54), followed by Indians with OR = 2.44 and 95% CI = (0.39–14.91) and Malays with OR = 2.23 and 95% CI = (0.23–2.75). As for the allele minor versus major, the Indians scored OR = 2.433 and 95% CI = (1.458–4.066), followed by the Chinese scoring OR = 2.137 and 95% CI = (3.634–1.280) and lastly the Malays scoring OR = 1.8 and 95% CI = (1.080–2.999). The same pattern of strong association in the dominant model was found where the Chinese had OR = 2.27 and 95% CI = (1.26–4.11), followed by the Indians having OR = 2.22 and 95% CI = (1.26–3.93) and the Malays having OR = 0.44 and 95% CI = (0.25–0.78). On the contrary, in the multiplicative model, the Indians showed the highest association with OR = 1.94 and 95% CI = (1.19–3.17), while the Malays with OR = 1.80 and 95% CI = (1.08–3.00) and the Chinese with OR = 1.80 and 95% CI = (1.08–2.99) showed the same strength of association.

## 4. Discussion

In this study, four polymorphisms of three different genes were tested to confirm variants that might be the risk factors that increase the susceptibility to DN among Malaysians. All four are related to oxidative stress and have significant relations with nephropathy development in all of the Malaysian subjects. The oxidative stress due to mitochondrial overproduction of ROS has been demonstrated to be the factor in the vascular pathobiology of diabetic microvascular complications [15]. Oxidative stress activates other related pathophysiologies in T2DM [22].

The activity of carnosinase was expected to be increased by *rs2346061* due to the location of the SNP in the regulatory or promoter region of *CNDP1* and *CNDP2*. SNP *rs2346061* alters the activity of carnosinase by controlling and modulating the expression of *CNDP1* and *CNDP2*. In this research, SNP *rs2346061* associated weakly with the additive model among the Indians. This finding is similar to the study of Ahluwalia et al. on T2DM patients in Sweden (OR = 1.25 and 95% CI = (1.1–1.4) in the additive model) [17]. On the contrary, however, Kurashige et al. [23] did not find any association in the Japanese women, and the same finding was also observed from a study on the African American T2DM patients [24]. Meanwhile, a study on type 1 diabetic Europeans also showed no association too [25]. In this study, the Malays and Chinese do not show any association, indicating different *rs2346061* effects among the ethnic groups. According to Ahluwalia et al. [17], SNP *rs2346061* had no association with kidney function but would only increase the risk of albuminuria and not the progression of kidney disease. This was explained by observing the neutral effect of *rs2346061* on eGFR. The variant did not directly affect the kidney function. SNP *rs2346061* is located at *CNDP1* which is primarily in distal tubules. *CNDP1* is expressed mainly in the brain (particularly in pyramidal cells of the hippocampus) and liver, not in the kidney [26]. As the association of *rs2346061* is quite weak in some population, sampling more samples would benefit the analysis because *rs2346061* also increases the expression of the carnosinase variant of 5 CTG repeats among the nephrotic samples which affect the overall eGFR value. The number of samples was calculated by assuming the strength of the variants is medium (OR > 2.5) when associated with nephropathy and that would give the error of at least 20%. This type of error occurred during sampling. Increasing the number of samples will minimize the error. The low number or frequency of allele carriers would require a larger number of samples. Now the distribution or exposure of the risk allele SNP *rs2346061* is known, and a suitable number of samples could be calculated correctly for future studies.

Diabetic patients with the lowest number of leucine repeats are less susceptible to DN. Individuals with homozygous or heterozygous allele for more than 5 leucine repeats have reduced serum carnosine concentration [27]. Carnosine (*β*-alanyl-L-histidine) has been reported to have served as an oxygen-free radical scavenger [28], natural ACE inhibitor, and cleave advanced glycation end product [29, 30]. These can only be achieved if the concentration level of renal carnosine is sustained at its protective level. Janssen et al. [31] had observed that the addition of carnosine in renal cell lines could markedly reduce the synthesis of matrix components and TGF*β*2 in renal cell lines, which thus prevents further development of nephropathy. Immunostaining techniques revealed and proved that *CNDP1* is present in the human kidney tissue. Carnosinase secretion encoded from the *CNDP1* gene is significantly greater in podocytes and renal parietal epithelial cells from the subjects with DN, with variants, more than 5 CTG repeats, compared with healthy renal tissue with variants 5 CTG repeats or less. The location of (CTG)*n* repeat is on the hydrophobic core of the signal peptide, and 5 CTG repeats provide a low signal, causing inefficiency for excretion compared to CTG with *n* > 5 repeats [32]. This study reports that *CNDP1* polymorphism distribution in three ethnic groups in Malaysia shows a significant association of reduced risk of 5/5 homozygous genotypes. In the control group, the frequency of 5/5 homozygous genotypes is very low: 6.2% in Malays, 8.4% in Chinese, and 15.4% in Indians. This may be the cause of the high occurrence of nephropathy in T2DM in the Malaysian population. The mutant allele carriers of the case are very high in Malaysian population with Malays = 88.9%, Chinese = 88.6%, and Indians = 69.8%, which may also explain the reason why 90% of the T2DM patients are having nephropathy. The lower frequency of 5-5 homozygous genotypes has led to higher carnosinase activity levels among Malaysians, therefore decreasing the carnosine level and resulting in oxidative stress and its effects. The lowest frequency of 5-5 homozygous genotypes ever reported was among the Japanese population <0.1% [23]. Chinese peritoneal dialysis patients were about 0.9% [33], and South Asian Surinamese was 23.0% [34]. From the Malaysian data given above, it is expected that the Indians are more protected compared to other races. The odds ratio of the Indians is the least in all the models except in the dominant model. Overall, the odds ratio among the races ranged from 1.70–2.46 caused by *D18S880* polymorphism and was considered as a moderate association or less risk. There was, however, no association in *D18S880* polymorphism among the African Americans [24, 34, 35], Scandinavians [17] for T2DM, and Europeans for T1DM [25], thus showing that there are other possible variants involved in the development of nephropathy in T2DM.

Normally, mitochondrial ROS is one of the defense mechanisms against bacteria in the human system [36]. The normal level of mitochondrial ROS needs to be maintained optimally by decreasing the excess which is scavenged by MnSOD for cell survival [37, 38]. MnSOD or SOD2 gene encodes for the iron/manganese superoxide dismutase family, a mitochondrial protein that forms a homotetramer and binds one manganese ion per subunit. This protein binds to the superoxide by-products of oxidative phosphorylation and converts them to hydrogen peroxide and diatomic oxygen. SNP *rs4880* C > T in this gene has been associated with T2DM nephropathy. The *rs4880* (V16A) is identified at the 16^th^ amino acid position of MnSOD [21], altering the structure of MnSOD. The C allele codes for a partial alpha-helix in the Ala variant to a beta-sheet but once substituted for T allele, it codes for a beta-sheet in the Val variant, causing less efficient transport of MnSOD into the mitochondrial matrix and becoming 30–40% weaker antioxidant for mitochondrial ROS [39]. In the hyperglycemic atmosphere, mitochondrial ROS is induced abundantly by activation of the electron transport system [40], causing an imbalance between endogenous prooxidative and antioxidative systems. As hyperglycemia persists, it can also contribute to the progression as well as initiation of diabetic microvascular complications. During this condition, the liver and kidney are the two organs that have high mitochondrial ROS levels raised abruptly, but the MnSOD level was observed to increase in the liver only. This may suggest that the mitochondrial oxidative reaction may cause oxidative damage in the kidney at an early stage of diabetes. The frequency of the genotype TT is very low: 3.2%, 2.1%, and 3.7% in Malays, Chinese, and Indians, from the controls. The frequency of T allele carriers in the case group is 22.5% among Malays, 21.8% among Chinese, and 23.8% among Indians, which does not seem to be significantly different between the groups (*p* > 0.05).

The association was demonstrated in all of the models for the Indians, but for the Malays, it was not significant in the recessive model, while among the Chinese, it was not significant in the dominant model. The Cochran–Armitage trend testing proves that the T allele behaved more towards being additive compared to the others, suggesting that the C allele has a reduced risk of T2DM nephropathy. In the additive model, the odds ratio showed the same moderate strength of association in the Malays and Indians but showed quite strong strength of association among the Chinese, as observed in single and double copy alleles in the forest plot with the estimates of odds ratios and 95% confidence intervals. A significant association was also observed among the Koreans [41], French [42], and Mexicans [43], but negative association was observed among the Slovenians (Caucasians) [44]. A meta-analysis found that *rs4880* showed a significant correlation between nephropathy in both T1DM and T2DM [45], suggesting that the C allele of a C47T polymorphism in the SOD2 gene had significant protective effects on the risk of DN.

In this research, there was a significant association among the Malaysian population in all models except for the recessive model among the Chinese and Indians. As for the Malays, all models showed association except in the dominant model. The additive model has the least *p* value from the Cochran–Armitage trend test, indicating the SNP was having the additive mode of inheritance. The Chinese had the strongest association. The frequencies of the T allele carrier in the nonnephrotic samples were only 13.5% to 14.5%, but the OR value is high suggesting a moderate-to-strong association of the variant to develop nephropathy among T2DM patients. This is because the various functions of *NOS3* are affected due to the missense resulted from Glu to Asp substitution at residue 298 of *NOS3*. The missense lowered NOS3 activity; thus, NO production is affected. The low level of NO fails to suppress oxidative stress, platelet aggregation, leukocyte adhesion, and smooth muscle cell proliferation, triggering many pathways which lead to the development of DN [46]. The T allele was found to be associated with T2DM nephropathy in most Asian populations such as the Japanese [47–49], Koreans [50], Indonesians [51], North Indians [52], and Tunisians [53]. On the contrary, Zintzaras et al. [54] found that the T allele is shown to have a weak association with T2DM patients but strong association with diabetes leading to severe nephropathy in East Asians. However, the GG genotype seemed to increase the risk in T2DM patients with chronic renal insufficiency among the South Indian population but not among the North Indians [55].

## 5. Conclusion

All four genes carnosinase (*CNDP1-D18S880* and -*rs2346061*), endothelial nitric oxide synthase (*NOS3*-*rs1799983*), and manganese superoxide dismutase (*MnSOD*-*rs4880*) with oxidative stress-related polymorphisms have significant effects on the development of nephropathy in Malaysian type 2 diabetes patients. The genes may, therefore, be considered as risk factors for Malaysian subjects who are predisposed to T2DM nephropathy although differing among the three races. Based on the least *p* value compared to the other models, the best model fit for *D18S880* was multiplicative for all the races: Malays (*p*=0.0004), Chinese (*p*=0.0019), and Indians (*p*=0.005), while for the other variants, the best model was additive.

## Figures and Tables

**Table 1:** Inclusion and exclusion criteria.

No.	Inclusion criteria of nephropathy group	Inclusion criteria of without nephropathy group	Exclusion criteria
1	Biologically unrelated	Biologically unrelated	Biologically related
2	Age onset ≥ 35 years	Age onset ≥ 35 years	Age onset ≤ 35 years
3	Diabetes duration ≥ 10 years	Diabetes duration ≥ 10 years	Diabetes duration ≤ 10 years
4	Fasting plasma glucose ≥ 7.0 mmol/L	Fasting plasma glucose ≥ 7.0 mmol/L	Normal fasting glucose level
5	Albumin excretion rate > 300 mg/24 h	Albumin excretion rate < 30 mg/24 h	Nondiabetic and normal rate
6	Albumin creatinine ratio is >3.5 mg/mmol for women and >2.5 mg/mmol for men	Albumin creatinine ratio is <3.5 mg/mmol for women and <2.5 mg/mmol for men	Patients without renal symptoms with a duration of <10 years of diabetes
7	ESRD of T2D patients	Non-ESRD of T2DM patients	Unclear of renal damage and ESRD or non-ESRD of T1DM patients
8	Glycated hemoglobin (HbA1c) > 6.6%	Glycated hemoglobin (HbA1c) ≤ 6.5%	Glycated hemoglobin (HbA1c) < 6.5%

ESRD = end-stage renal disease; T2DM = type 2 diabetes mellitus; T1DM = type 1 diabetes mellitus.

**Table 2:** Sequence of primers and size of the PCR products used for the genotyping.

No.	SNP	Forward	Reverse	PCR products (bp)	*T*_m_ (°C)
1	*rs2346061*	ACGTTGGATGTGATGTTCTCCCTGTGTATG	ACGTTGGATGATGGACCCCTGATTACACAC	100	46.8
2	*rs4880*	ACGTTGGATGTTCTGCCTGGAGCCCAGATA	ACGTTGGATGGGCTGTGCTTTCTCGTCTTC	93	54.5
3	*rs1799983*	ACGTTGGATGTGCATTCAGCACGGCTGGAC	ACGTTGGATGGGGCAGAAGGAAGAGTTC	119	59.9

**Table 3:** Clinical and demographic characteristics of type 2 diabetic patient samples.

Characteristic	Malays (*n* = 227)			Chinese (*n* = 203)			Indians (*n* = 222)		
	With nephropathy	Without nephropathy	*p* value	With nephropathy	Without nephropathy	*p* value	With nephropathy	Without nephropathy	*p* value
Number of samples	131 (57.7%)	96 (42.3%)	—	108 (53.2%)	95 (46.8%)	—	86 (38.7%)	136 (61.3%)	—
Duration of diabetes (years)	16.1 ± 6.8	15.5 ± 5.1	0.061	16.6 ± 6.7	16.8 ± 6.6	0.060	17 ± 5.1	16.8 ± 5.0	0.070
Albumin excretion rate (g/24 h)	1363.18 ± 136.00	25.83 ± 2.10	<0.005^*∗*^	1952.50 ± 144.30	23.00 ± 5.70	<0.005^*∗*^	1756 ± 155.44	26.67 ± 1.9	<0.005^*∗*^
Glycated hemoglobin (%)	8.67 ± 2.34	9.07 ± 1.97	0.087	9.19 ± 2.31	9.45 ± 2.56	0.176	7.79 ± 2.08	8.30 ± 1.63	0.192
Fasting blood glucose (mmol/L)	9.91 ± 3.3	9.86 ± 3.70	0.327	9.60 ± 2.10	8.6 ± 3.70	0.251	9.8 ± 0.137	8.78 ± 3.69	0.137
Total cholesterol (mmol/L)	6.42 ± 1.37	4.84 ± 1.01	0.002^*∗*^	6.58 ± 1.19	4.26 ± 1.13	0.003^*∗*^	6.48 ± 1.42	4.74 ± 1.23	0.004^*∗*^
HDL cholesterol (mmol/L)	1.02 ± 0.39	1.22 ± 0.26	0.001^*∗*^	1.01 ± 0.24	1.11 ± 0.24	0.003^*∗*^	1.16 ± 0.25	1.29 ± 0.26	0.001^*∗*^
LDL cholesterol (mmol/L)	2. 45 ± 1.25	2.82 ± 1.10	0.732	2.69 ± 1.05	2.53 ± 0.90	0.491	2.61 ± 1.25	2.60 ± 1.06	0.626
Triglycerides (mmol/L)	1.85 ± 0.80	2.07 ± 1.81	0.168	1.72 ± 0.64	1.36 ± 0.80	0.844	1.55 ± 0.67	1.87 ± 0.87	0.393

^*∗*^ *p* < 0.05 shows a significant difference.

**Table 4:** Differences in the frequencies of allele distribution among the races.

SNP	Control							Case						
	Malays		Chinese		Indians		*p* value (*χ* ^2^, df = 2)	Malays		Chinese		Indians		*p* value (*χ* ^2^, df = 2)
*CNDP1*	*A* = 161	*C* = 31	*A* = 140	*C* = 50	*A* = 187	*C* = 41	**0.028** ^*∗*^	*A* = 213	*C* = 49	*A* = 161	*C* = 49	*A* = 94	*C* = 40	**0.042** ^*∗*^
*rs2346061*	(83.9)	(16.1)	(73.7)	(26.3)	(82.0)	(18.0)	7.1	(81.3)	(18.7)	(76.7)	(23.3)	(70.1)	(29.9)	6.32
*CNDP1*	5 = 45	6 = 147	5 = 43	6 = 147	5 = 118	6 = 154	**<0.05** ^*∗*^	5 = 29	6 = 233	5 = 27	6 = 209	5 = 52	6 = 120	<**0.001** ^*∗*^
*D18S880*	(23.4)	(76.6)	(22.6)	(77.4)	(43.4)	(56.6)	30.51	(11.1)	(88.9)	(11.4)	(88.6)	(30.2)	(69.8)	34.1
*MnSOD*	*C* = 166	*T* = 26	*C* = 167	*T* = 23	*C* = 232	*T* = 38	0.920	*C* = 203	*T* = 59	*C* = 153	*T* = 47	*C* = 131	*T* = 41	0.942
*rs4880*	(86.5)	(13.5)	(87.9)	(12.1)	(85.93)	(14.07)	0.01	(77.5)	(22.5)	(78.2)	(21.8)	(76.2)	(23.8)	0.12
*NOS3*	*G* = 166	*T* = 26	*G* = 166	*T* = 26	*G* = 236	*T* = 38	0.842	*G* = 202	*T* = 57	*G* = 165	*T* = 51	*G* = 131	*T* = 41	0.878
*rs1799983*	(86.5)	(13.5)	(86.5)	(13.5)	(85.5)	(14.5)	0.04	(78.0)	(22.0)	(76.4)	(23.6)	(76.2)	(23.8)	0.26

^*∗*^ *p* < 0.05 indicates the significant difference in allele distribution in the population.

**Table 5:** Hardy–Weinberg equilibrium test for the controls and cases.

SNP	Malays						Chinese						Indians					
	Control			Statistic			Control			Statistic			Control			Statistic		
	Major/major	Major/minor	Minor/minor	*χ* ^2^	*p* value	df	Major/major	Major/minor	Minor/minor	*χ* ^2^	*p* value	df	Major/major	Major/minor	Minor/minor	*χ* ^2^	*p* value	df
*Control*																		
*CNDP1*	AA = 68	CA = 25	CC = 3	0.140	0.7083	1	AA = 53	CA = 34	CC = 8	3.370	0.0664	1	AA = 74	CA = 37	CC = 2	1.185	0.2763	1
*rs2346061*	(70.8)	(26.0)	(3.1)				(55.8)	(35.8)	(8.4)				(65.5)	(32.7)	(1.8)			
*CNDP1*	5-5 = 6	6-5 = 33	6-6 = 57	0.171	0.6792	1	5-5 = 8	6-5 = 27	6-6 = 60	3.372	0.0663	1	5-5 = 21	6-5 = 76	6-6 = 39	2.574	0.1086	1
*D18S880*	(6.2)	(34.4)	(59.4)				(8.4)	(28.4)	(63.2)				(15.4)	(55.9)	(28.7)			
*MnSOD*	CC = 73	TC = 20	TT = 3	1.168	0.2798	1	CC = 74	TC = 19	TT = 2	0.439	0.5076	1	CC = 102	TC = 28	TT = 5	2.740	0.0979	1
*rs4880*	(76.0)	(20.8)	(3.2)				(77.9)	(20.0)	(2.1)				(75.6)	(20.7)	(3.7)			
*NOS3*	GG = 71	GT = 24	TT = 1	0.439	0.5076	1	GG = 71	GT = 24	TT = 1	1.904	0.1676	1	GG = 101	GT = 34	TT = 2	0.206	0.6499	1
*rs1799983*	(74.0)	(33.0)	(1.0)				(74.0)	(25.0)	(1.0)				(73.7)	(24.8)	(1.5)			

*Case*																		
*CNDP1*	AA = 87	CA = 39	CC = 5	0.057	0.8102	1	AA = 61	CA = 39	CC = 5	0.152	0.6958	1	AA = 33	CA = 30	CC = 4	0.697	0.4039	1
*rs2346061*	(66.4)	(29.8)	(3.8)				(58.1)	(36.2)	(8.9)				(49.2)	(44.8)	(6.0)			
*CNDP1*	5-5 = 3	6-5 = 23	6-6 = 105	1.533	0.2156	1	5-5 = 3	6-5 = 21	6-6 = 94	1.749	0.1860	1	5-5 = 4	6-5 = 44	6-6 = 38	3.895	0.0484	1
*D18S880*	(2.3)	(17.6)	(80.1)				(2.5)	(17.8)	(79.7)				(4.7)	(51.2)	(41.1)			
*MnSOD*	CC = 79	TT = 45	TT = 7	0.032	0.8582	1	CC = 66	TC = 37	TT = 5	0.004	0.9490	1	CC = 51	TC = 29	TT = 6	0.4373	0.5084	1
*rs4880*	(60.3)	(34.3)	(5.4)				(61.1)	(34.3)	(4.6)				(59.3)	(33.7)	(5.8)			
*NOS3*	GG = 74	GT = 54	TT = 3	3.668	0.0555	1	GG = 60	GT = 45	TT = 3	0.9091	0.3404	1	GG = 48	GT = 35	TT = 3	1.256	0.2625	1
*rs1799983*	(56.5)	(41.2)	(2.3)				(55.6)	(41.7)	(2.7)				(58.8)	(40.7)	(3.5)			

*p* > 0.05 shows consistency with HWE. Genotype data are presented as a number of subjects (%).

**Table 6:** Association of polymorphism in T2DM with and without nephropathy.

*CNDP1 χ* ^2^ *rs2346061*		Malays				Chinese				Indians			
		Multiplicative model		Dominant model	Recessive model	Multiplicative model		Dominant model	Recessive model	Multiplicative model		Dominant model	Recessive model
		Genotype (df = 2)	Allele (df = 1)	Major/major vs. others (df = 1)	Minor/minor vs. others (df = 1)	Genotype (df = 2)	Allele (df = 1)	Major/major vs. others (df = 1)	Minor/minor vs. others (df = 1)	Genotype (df = 2)	Allele (df = 1)	Major/major vs. others (df = 1)	Minor/minor vs. others (df = 1)
*CNDP1*	*χ* ^2^	—	0.499	0.566	*—*	1.242	0.476	0.325	1.099	—	6.844	4.597	—
*rs2346061*	*p*	0.7883	0.4799	0.4518	0.5410	0.5374	0.4902	0.5686	0.2944	**0.0488** ^*∗*^	**0.0089** ^*∗*^	**0.0320** ^*∗*^	0.1390
*CNDP1*	*χ* ^2^	11.894	12.425	2.282	11.704	8.141	9.600	3.714	7.157	9.318	7.711	6.138	6.138
*D18S880*	*p*	**0.0026** ^*∗*^	**0.0004** ^*∗*^	0.1302	**0.0006** ^*∗*^	**0.0171** ^*∗*^	**0.0019** ^*∗*^	0.0540	**0.0075** ^*∗*^	**0.0095** ^*∗*^	**0.0055** ^*∗*^	**0.0132** ^*∗*^	**0.0132** ^*∗*^
*MnSOD*	*χ* ^2^	6.203	5.868	6.201	0.647	—	6.603	0.967	8.423	15.235	16.301	10.689	7.238
*rs4880*	*p*	**0.0450** ^*∗*^	**0.0154** ^*∗*^	**0.0128** ^*∗*^	0.4210	**0.0380** ^*∗*^	**0.0101** ^*∗*^	0.3254	**0.0037** ^*∗*^	**49.2 × 10** ^**−5**^^*∗*^	**5.4 × 10** ^**−5**^^*∗*^	**0.0011** ^*∗*^	**0.0071** ^*∗*^
*NOS3*	*χ* ^2^	—	5.263	0.499	—	—	6.731	7.490	—	—	7.204	7.642	—
*rs1799983*	*p*	**0.0158** ^*∗*^	**0.0218** ^*∗*^	0.4799	0.4350	**0.0146** ^*∗*^	**0.0095** ^*∗*^	**0.0062** ^*∗*^	0.6667	**0.0174** ^*∗*^	**0.0073** ^*∗*^	**0.0057** ^*∗*^	**0.4518**

^*∗*^ *p* < 0.05 indicates an association of polymorphisms and disease in a different mode of inheritance.

**Table 7:** Cochran–Armitage trend testing.

		Malays				Chinese				Indians			
		Multiplicative (df = 1)	Additive (df = 1)	Dominant (df = 1)	Recessive (df = 1)	Multiplicative (df = 1)	Additive (df = 1)	Dominant (df = 1)	Recessive (df = 1)	Multiplicative (df = 1)	Additive (df = 1)	Dominant (df = 1)	Recessive (df = 1)
*CNDP1* *rs2346061*	*χ* ^2^ *p*	—	—	—	—	—	—	—	—	4.233 **0.0396** ^*∗*^	0.944 0.3313	0.608 0.4355	0.1887 0.6640

*CNDP1* *D18S880*	*χ* ^2^ *p*	12.425 **0.0004** ^*∗*^	−8.3667 —	11.704 0.0006^*∗*^	2.282 0.1309	9.600 0.0019^*∗*^	−8.042 —	7.157 0.0074^*∗*^	3.714 0.0540	7.711 0.0055^*∗*^	−10.552 —	5.594 0.0180^*∗*^	6.138 0.0132^*∗*^

*MnSOD* *rs4880*	*χ* ^2^ *p*	5.868 0.0154^*∗*^	6.569 **0.0104** ^*∗*^	0.647 0.4210	6.201 0.0128^*∗*^	6.603 0.0102^*∗*^	7.336 **0.0068** ^*∗*^	0.967 0.3254	6.652 0.0099^*∗*^	6.823 0.0090^*∗*^	7.208 **0.0073** ^*∗*^	1.189 0.2755	6.514 0.0107^*∗*^

*NOS3* *rs1799983*	*χ* ^2^ *p*	5.263 0.0217^*∗*^	7.879 **0.0050** ^*∗*^	0.499 0.4799	7.328 0.0068^*∗*^	6.731 0.0220^*∗*^	8.323 **0.0039** ^*∗*^	0.797 0.3720	7.490 0.0062^*∗*^	7.204 0.0073^*∗*^	8.542 **0.0035** ^*∗*^	0.992 0.3193	7.642 0.0057^*∗*^

The mode of inheritance is best presented with the least *p* value^*∗*^.

## Data Availability

The data used to support the findings of this study are included within the article.

## Supplementary Materials

Supplementary Materials Table S1: genotype distribution and frequencies in cases and controls. Table S2: allele distribution and frequencies in cases and controls.
