# Supplementary material for: CNDP1, NOS3, and MnSOD Polymorphisms as Risk Factors for Diabetic Nephropathy among Type 2 Diabetic Patients in Malaysia
Source: J Nutr Metab. 2019 Jan 3;2019:8736215. doi: 10.1155/2019/8736215 (PMC6335667; doi:10.1155/2019/8736215)
Supplement: Supplementary Materials — Table S1: genotype distribution and frequencies in cases and controls. Table S2: allele distribution and frequencies in cases and controls. [file 8736215.f1.docx]

Table 1

Genotype distribution and frequencies in case and control

| ***SNP*** | **Malay** | | | | | | ***Chinese*** | | | | | | ***Indian*** | | | | | |
| --- | --- | --- | --- | --- | --- | --- | --- | --- | --- | --- | --- | --- | --- | --- | --- | --- | --- | --- |
|  | **Case** | | | **Control** | | | ***Case*** | | | ***Control*** | | | ***Case*** | | | ***Control*** | | |
|  | major/ major | major/ minor | minor/ minor | major/ major | major/ minor | minor/ minor | major/ major | major/ minor | minor/ minor | major/ major | major/ minor | minor/ minor | major/ major | major/ minor | minor/ minor | major/ major | major/ minor | minor/ minor |
| *CNDP1 rs2346061* | AA=87 (66.4) | CA=39 (29.8) | CC=5 (3.8) | AA=68 (70.8) | CA=25 (26.0) | CC=3 (3.1) | AA=61 (58.1) | CA=39 (36.2) | CC=5 (8.9) | AA=53 (55.8) | CA=34 (35.8) | CC=8 (8.4) | AA=33 (49.2) | CA=30 (44.8) | CC=4 (6.0) | AA=74 (65.5) | CA=37 (32.7) | CC=2 (1.8) |
| *CNDP1* | 5-5=3 | 6-5=23 | 6-6=105 | 5-5=6 | 6-5=33 | 6-6=57 | 5-5=3 | 6-5=21 | 6-6=94 | 5-5=8 | 6-5=27 | 6-6=60 | 5-5=4 | 6-5=44 | 6-6=38 | 5-5=21 | 6-5=76 | 6-6=39 |
| *D18S880* | (2.3) | (17.6) | (80.1 ) | (6.2 ) | ( 34.4) | ( 59.4) | (2.5) | (17.8) | (79.7) | (8.4) | (28.4) | (63.2) | (4.7) | (51.2) | (41.1) | ( 15.4) | (55.9 ) | ( 28.7) |
| *MnSOD*  *rs4880* | CC=79 (60.3) | TC=45 (34.3) | TT=7 (5.4) | CC=73 (76.0) | TC=20 (20.8) | TT=3 (3.2) | CC=66 (61.1) | TC=37 (34.3) | TT=5 (4.6) | CC=74 (77.9) | TC=19 (20.0) | TT=2 (2.1) | CC=51 (59.3) | TC=29 (33.7) | TT=6 (5.8) | CC=102 (75.6) | TC=28 (20.7) | TT=5 (3.7) |
| *NOS3 rs1799983* | GG=74 (56.5) | GT=54 (41.2) | TT=3 (2.3) | GG=71 (74.0 ) | GT=24 (25.0 ) | TT=1 (1.0) | GG=60 ( 55.6) | GT=45 ( 41.7) | TT=3 (2.7) | GG=71 ( 74.0) | GT=24 (25.0) | TT=1 ( 1.0) | GG=48 (58.8) | GT=35 (40.7) | TT=3 (3.5) | GG=101 (73.7) | GT=34 (24.8) | TT=2 (1.5) |

Genotype data are presented as number of subjects (%).

Table 2

Allele distribution and frequencies in case and control

| **SNP** | **Malay** | | | | **Chinese** | | | | **Indian** | | | |
| --- | --- | --- | --- | --- | --- | --- | --- | --- | --- | --- | --- | --- |
|  | **Case** | | **Control** | | **Case** | | **Control** | | **Case** | | **Control** | |
| *CNDP1 rs2346061* | A=213 (81.3) | C=49 (18.7) | A=161 (83.9) | C=31 (16.1) | A=161 (76.7) | C=49 (23.3) | A=140 (73.7) | C=50 (26.3) | A=94 (70.1) | C= 40  (29.9) | A=187 (82.0) | C= 41  (18.0) |
| *CNDP1* | 5=29 | 6=233 | 5=45 | 6=147 | 5=27 | 6=209 | 5=43 | 6=147 | 5= 52 | 6=120 | 5=118 | 6=154 |
| *D18S880* | (11.1) | (88.9) | (23.4) | (76.6) | (11.4) | (88.6) | (22.6) | (77.4) | (30.2) | (69.8) | (43.4) | (56.6) |
| *MnSOD*  *rs4880* | C=203 (77.5) | T=59 (22.5) | C=166 (86.5) | T=26 (13.5) | C=153 (78.2) | T=47 (21.8) | C=167 (87.9) | T=23 (12.1) | C=131 (76.2) | T=41 (23.8) | C=232 (85.93) | T=38 (14.07) |
| *NOS3 rs1799983* | G=202 (78.0) | T=57 (22.0) | G=166 (86.5) | T=26 (13.5) | G=165 (76.4) | T=51 (23.6) | G=166 (86.5) | T=26 (13.5) | G=131 (76.2) | T=41 (23.8) | G=236 (85.5) | T=38 (14.5) |

Allele data are presented as a number of subjects (%).
